# Hernio-abdominoplasty with or without Scarpa’s Fascia Preservation for Ventral Hernia and Abdominal Wall Deformity

**DOI:** 10.1097/GOX.0000000000002302

**Published:** 2019-07-05

**Authors:** Mahmoud Eltantawy, Ayman Elshobaky, Waleed Thabet, Sameh Emile, Mohammed El-Said, Mohamed Taher Elshobaky, Sabry Ahmed Mahmoud

**Affiliations:** From the Department of General Surgery, Mansoura University Hospitals, Mansoura University, Mansoura, Egypt.

## Abstract

Scarpa’s fascia preservation plays a great role in decreasing the volume of wound drainage and reducing seroma after abdominoplasty. This study aimed to assess the role of Scarpa’s fascia preservation in patients with ventral hernias associated with abdominal wall deformity who underwent concomitant hernio-abdominoplasty in terms of early and late postoperative outcome and quality of life.

**Methods:**

Patients with ventral hernia and abdominal wall deformity underwent combined hernio-abdominoplasty. Patients were randomly allocated to 1 of 2 equal groups: group I underwent Scarpa’s fascia preserving hernio-abdominoplasty and group II underwent hernio-abdominoplasty with removal of Scarpa’s fascia. Volume of drainage, time to remove drains, return to work, and complications were recorded.

**Results:**

Fifty patients (49 female) were included to the study. Both groups had comparable operation time, pain score, and complication rate (24% versus 40%, *P* = 0.36). The mean total volume of postoperative drainage was significantly lower in group I than group II (686 ± 183.5 versus 1410.8 ± 371.6 ml; *P* < 0.0001). Group I had earlier drain removal (11.6 ± 1.9 versus 20.5 ± 4.2 days, *P* < 0.0001) and earlier return to work (16.4 ± 2.3 versus 23.3 ± 3.8 days, *P* < 0.0001) than group II. There were no recorded cases of hematoma or hernia recurrence after repair.

**Conclusion:**

Scarpa’s fascia preservation in combined ventral hernia repair and abdominoplasty was associated with significantly lower volume of postoperative drainage, earlier removal of drains, and similar recurrence rate to hernio-abdominoplasty with removal of Scarpa’s fascia.

## INTRODUCTION

Ventral abdominal hernia is a common surgical condition that accounts for approximately 25% of all abdominal hernias.^1^ Obesity is a major contributing factor to abdominal wall hernias; it is also associated with significant laxity of the skin and abdominal wall fascia.^2–4^

Ventral hernias are associated with marked abdominal wall laxity and redundancy in many patients which warrants concomitant repair of the abdominal wall defects and correction of the musculoaponeurotic laxity. Repair of ventral hernias combined with abdominoplasty has been described to improve the contour deformity of the whole musculofascial layer, especially in the waist area.^5^

One of the most frequent complications after both ventral hernia repair and abdominoplasty is seroma formation, with a reported incidence reaching up to 20%.^6^ To avoid seroma formation, subcutaneous drains are usually used; however, long-term drainage is not advisable, as it may increase patients’ discomfort.^7–10^

Preservation of Scarpa’s fascia helps preserve the arterio-veno-lymphatic system and improve the reabsorption of the fluid released from interstitial spaces.^11^ A prospective trial^12^ reported a significant decrease in the volume of wound drainage and earlier removal of drains in patients who underwent Scarpa’s fascia preserving abdominoplasty.

The present study aimed to assess the role of Scarpa’s fascia preservation in patients with ventral hernias associated with abdominal wall laxity and deformity who underwent concomitant on-lay prosthetic repair of ventral hernia and abdominoplasty in terms of volume of drainage, time to drain removal, complications, recurrence of hernia, and quality of life. Abdominal wall deformity was defined as excess skin and subcutaneous tissue associated with laxity of the abdominal wall musculature.

## METHODS

### Study Design

This was a randomized, single-blinded controlled trial (NCT03721575) on patients with ventral hernias and abdominal wall deformity who underwent on-lay mesh hernioplasty and abdominoplasty in the General Surgery Department, Mansoura University Hospital in the period of January 2016 to January 2018. Ethical approval from the institutional review board of Mansoura Faculty of Medicine was obtained.

### Eligibility Criteria

Patients included to the trial were adult patients below 60 years with ventral hernias and abdominal wall deformities of class III or IV according to Pitanguy’s classification of abdominal deformities.^13^

Only patients with body mass index (BMI) ≤40 kg/m^2^ and American Society of Anesthesiologists class I-II were included.

We excluded patients with (1) major abdominal wall defect warranting abdominal wall reconstruction; (2) recurrent incisional hernias after mesh hernioplasty; (3) complicated hernias defined as inflamed, incarcerated, and strangulated hernias; and (4) heavy smokers who smoke ≥25 cigarettes per day,^14^ patients with uncontrolled chest problems, uncontrolled diabetes mellitus, or coagulopathy.

### Random Sequence Generation and Blinding

Patients were randomly allocated to 1 of 2 equal groups: group I underwent Scarpa’s fascia preserving hernio-abdominoplasty and group II (control group) underwent classical hernio-abdominoplasty with removal of Scarpa’s fascia. Both groups underwent on-lay mesh repair of the ventral hernia.

Randomization was conducted by an online software (www.randomization.com). Allocation concealment was undertaken by sealed envelope method. The study was single-blinded, as the patients were aware of the nature of the study, yet not aware of the group they were allocated to. The operating surgeons were aware of treatment group allocations and the nature of the study.

### Preoperative Assessment

Patients were carefully assessed before surgery for abdominal wall deformity, skin laxity, excess adiposity, and muscle weakness according to Pitanguy’s classification.^13^ The waist circumference was measured as midway between the top of the iliac crests and the lower ribs while standing with the abdomen relaxed.^15^

### Preoperative Preparations

Written informed consents to participate in the trial were obtained from the patients before enrollment to the study. Patients with high risk for thromboembolism according to Geneva risk score for venous thromboembolism^16^ were administered a single subcutaneous injection of low molecular weight heparin (Enoxaprin 40 IU) at the night before surgery.

Preoperative pictures were taken in the anterior and lateral views in the anatomical position to compare with postoperative results. The intended sites of surgical incisions were marked while the patient was standing according to Le Louarn.^17^

### Surgical Technique

Procedures were done under general anesthesia. Two grams of ceftriaxone were given on induction. Classic abdominoplasty without Scarpa’s preservation was done according to Regnault,^18^ whereas Scarpa’s fascia preservation was done according to Le Louarn.^17^ The procedures were performed by a team of general surgeons that included one of the study authors (A.E.) who had prior training and experience in plastic surgery.

**Fig. 1.**
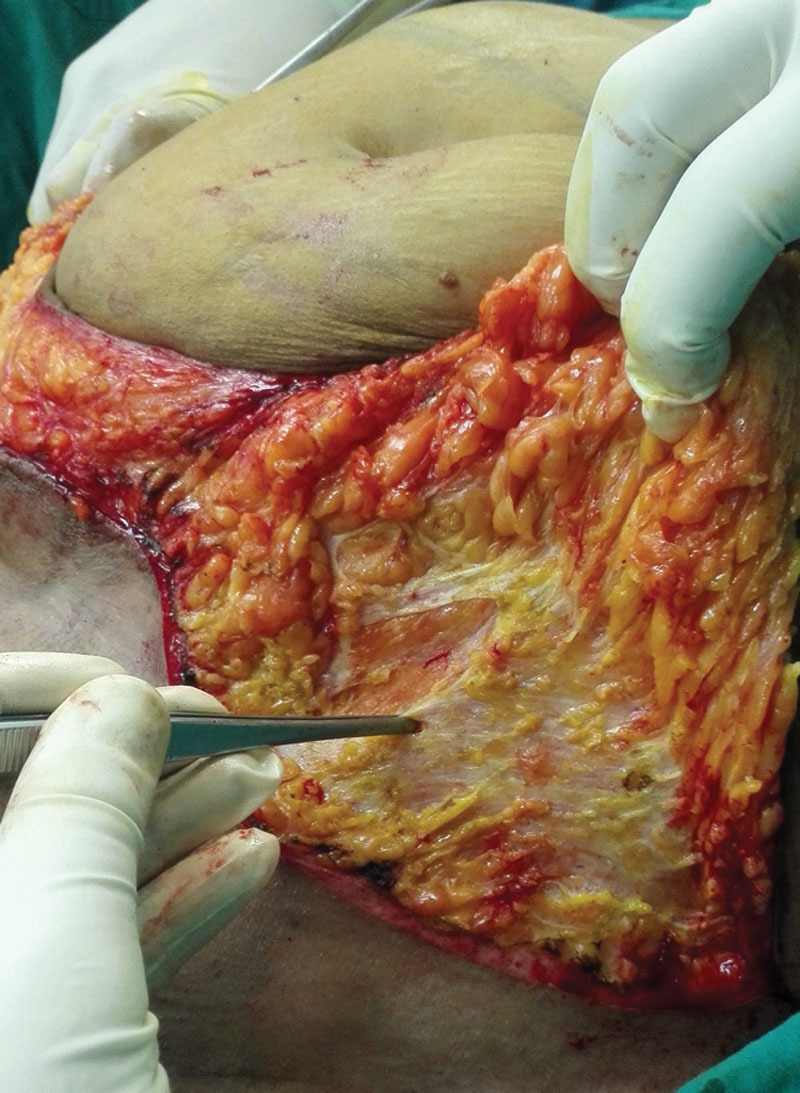
Dissection above the Scarpa’s fascia in group I.

Lower transverse incision was made first by scalpel, then deepened down to the external oblique aponeurosis and rectus sheath using electrocautery. Dissection was deepened laterally down to the Scarpa’s fascia and the flap was dissected just above Scarpa’s fascia in group I (Fig. 1), whereas in group II the dissection was undertaken above the external oblique aponeurosis, elevating the Scarpa’s fascia with skin flap.

**Fig. 2.**
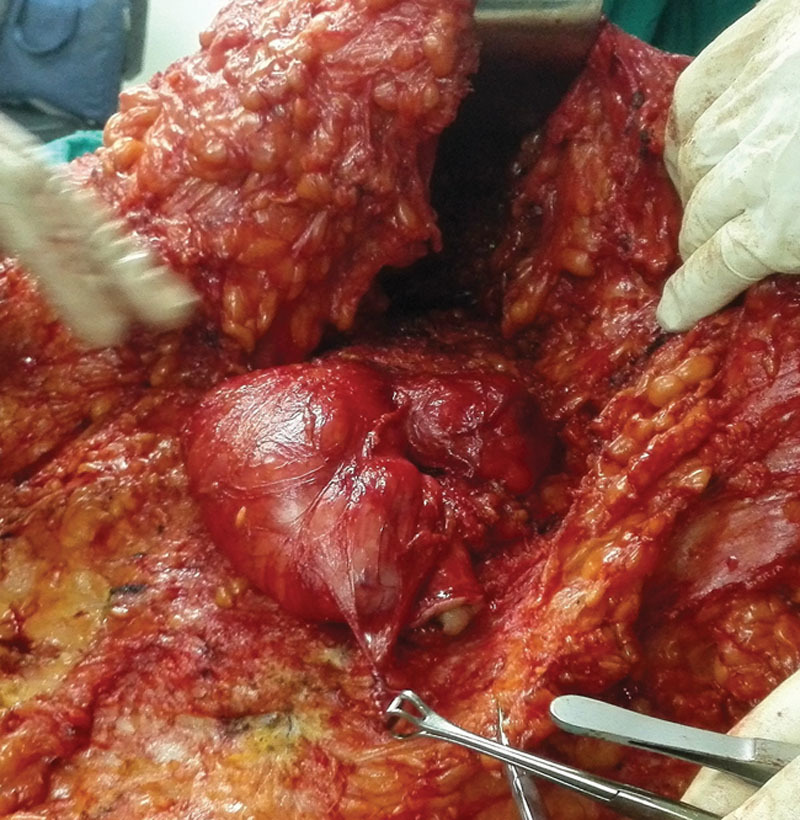
Dissection of the ventral hernia sac.

The flap was elevated till the level of the umbilicus, then an inverted V-shaped periumbilical incision was made to separate the umbilicus from the surrounding skin and the whole umbilical stalk was dissected using scissors down to the level of the anterior rectus sheath. The lower part of the upper abdominal flap below the umbilicus was split longitudinally to facilitate subsequent flap elevation above the level of the umbilicus then the hernia sac was dissected off the flap (Fig. 2).

**Fig. 3.**
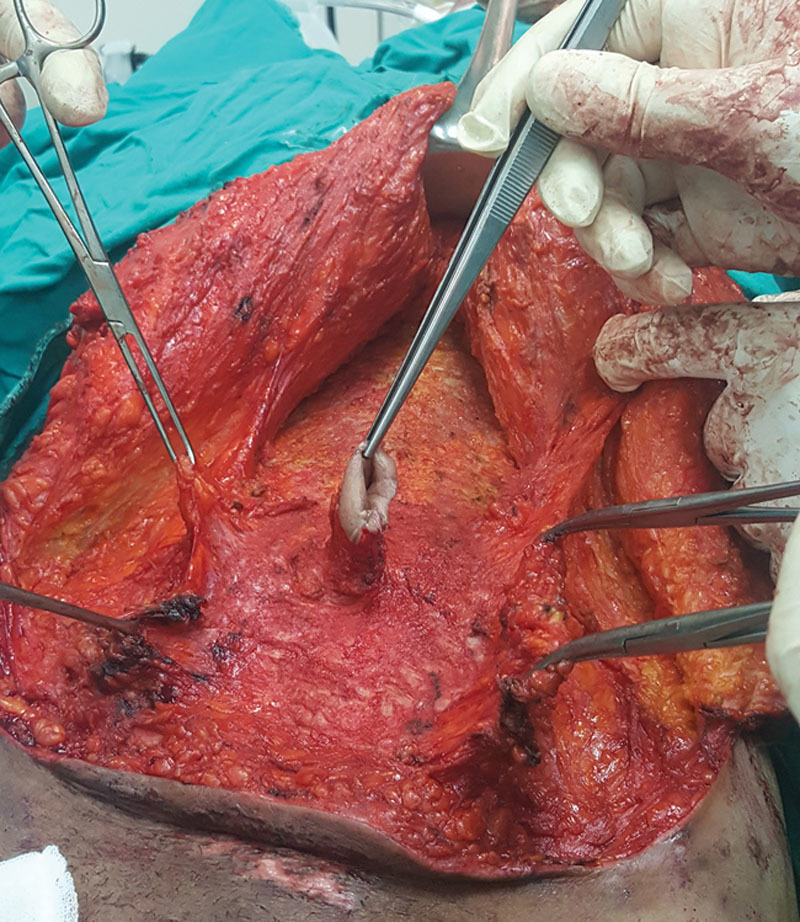
Supraumbilical dissection of the upper flap along with the Scarpa’s fascia immediately above the anterior rectus sheath.

The central part of the flap and the Scarpa’s fascia was elevated above the umbilicus using electrocautery till the xiphoid process, dissecting immediately above the anterior rectus sheath (Fig. 3). The dissection extended laterally, above the Scarpa’s fascia in group I, without exceeding the level of costal margins to preserve blood supply from the lateral intercostal, subcostal, and lumbar vessels. The lower flap was further elevated till the level of symphysis pubis.

**Fig. 4.**
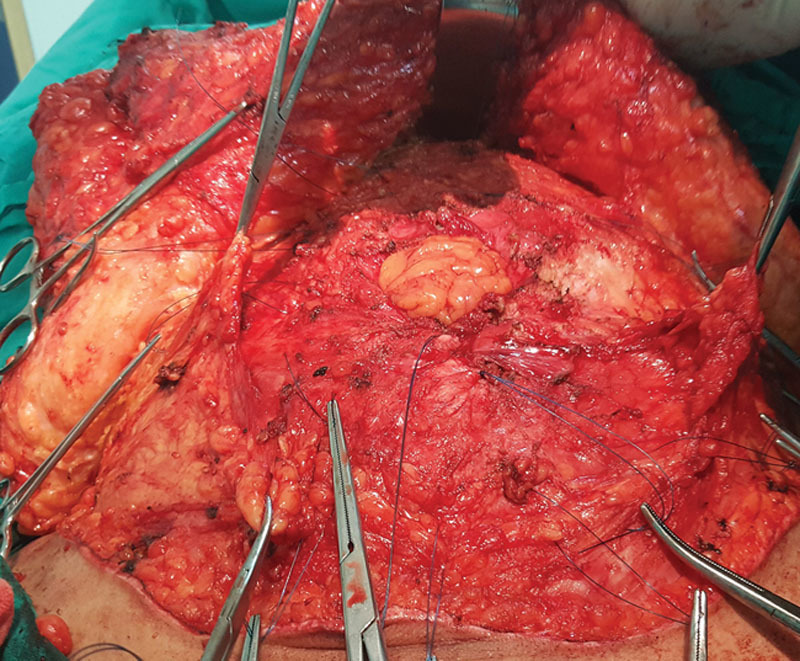
Pre-taken full-thickness polyprolene suture used to fixate the mesh.

After complete flap elevation, the hernia sac was opened and the contents were reduced back to the peritoneal cavity. Pre-taken full-thickness polypropylene 1 sutures were taken before closure of the defect (Fig. 4) about 5 cm from the edges of the defect. After all pre-taken sutures were taken, the defect was closed by continuous polyprolene 1 suture.

**Fig. 5.**
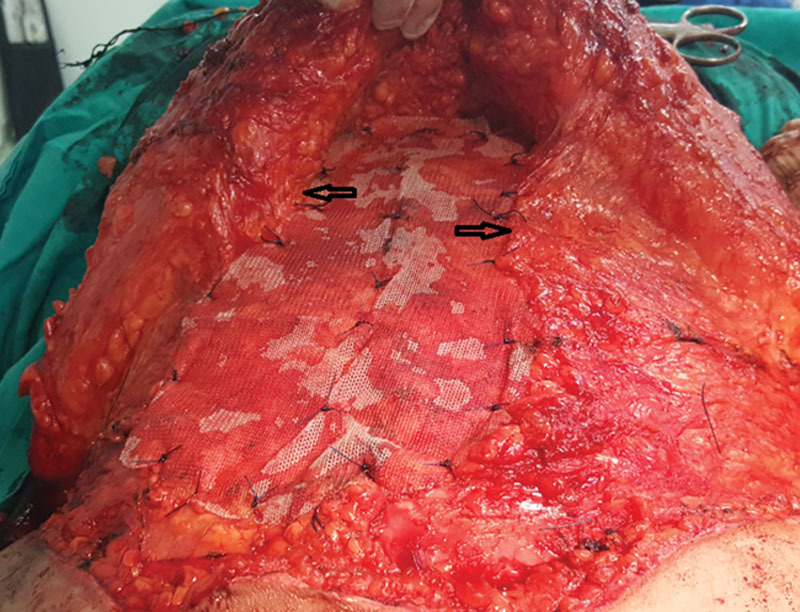
Fixation of the polyprolene mesh and medial advancement of the Scarpa’s fascia over the peripheral parts of the mesh (black arrows) in group I.

The linea alba was plicated using monofilament polyamide loop 1 suture starting from the xiphoid process in a downward direction reaching just above the umbilicus, then continued downward to the symphysis pubis, making sure that the closed abdominal defect was not included in the midline imbrication. Afterward, a microporous, heavy-weight, uncoated polypropylene mesh was placed above the rectus sheath using the on-lay technique (Fig. 5) with opening for the umbilical stalk if it was preserved, then the threads of the pre-taken sutures were inserted into the pores of the mesh and were tied to secure the mesh in place. The size of the mesh varied according to the size of the abdominal wall defect, making sure that the mesh would extend for 5 cm all around the defect.

In group I, the central part of Scarpa’s fascia, where the mesh is to be placed, was excised, whereas the lateral parts of the Scarpa’s fascia and sub-Scarpal tissue in the infraumbilical region were preserved. The lateral edges of the Scarpa’s fascia were advanced medially for 2–3 cm to cover the peripheral parts of the mesh and then were fixated to the anterior rectus sheath by 2/0 polyglactin sutures (Fig. 5). No progressive tension sutures were placed before closure. The upper flap was pulled downward and the excess skin was marked for subsequent excision in a symmetrical fashion.

The location of the new umbilicus was marked midway between the xiphoid process and symphysis pubis. An inverted V-shaped incision was made and the umbilicus was brought outside the incision and sutured using 4/0 polypropylene sutures. In the cases where the umbilical stalk was excised, an artificial umbilicus was created.

After adequate hemostasis was confirmed, 2 passive tube drains of size 24 Fr were brought out at the lateral edges of the wound and the wound was closed in 2 layers; Scarpa’s fascia and deep dermis then the skin using subcuticular 2/0 polypropylene sutures.

### Follow-up

Follow-up was done in the outpatient clinic at 4, 8, 14, 21, and 30 days postoperatively then every 3 months for 1 year.

The wound was inspected for infection, hematoma, or dehiscence. Wound healing and time to stitch removal were recorded. Pain was evaluated with Visual Analog Scale from 0 to 10 where 0 implied no pain and 10 indicated the worse severe pain. Patients were asked to record the volume of drain output on daily basis and the output was assessed by the investigators with regard to the amount and quality during follow-up visits until the drains were removed. Drains were removed when their output was below 30 ml/d.

At 1 month after surgery, the abdominal scar, quality of life, and drain output were assessed and photographs of the abdominal scar were taken. During follow-up, patients were assessed regarding any abdominal wall deformity, excess skin, abdominal wall sensation, body contour, recurrence of hernia, and quality of life as assessed by the Carolinas equation for quality of life.^19^

After drain removal, seroma was evaluated in the next visit. Clinical signs of seroma included swelling, discomfort, erythema, pain, and skin edema. Radiologic assessment by ultrasonography was done for clinically suspected seromas.

### Outcomes of the Trial

The primary outcome of the study was the total volume of drain output in milliliters. Secondary outcomes included the mean volume of drain output at 2, 4, 8, and 11 days after surgery, time to remove drains, operation time, hospital stay, viability of the flap and umbilicus, and complications including recurrence, postoperative pain, waist circumference, patients’ satisfaction, and quality of life.

### Sample Size Calculation

The sample size of the study was calculated by a power analysis of the primary endpoint of the study (total volume of drain output in ml) using online software (www.clincalc.com).

In light of the results of a previous trial^12^ that found the total volume of drain output to be 210 ml in the Scarpa’s preserving group and 609.2 ± 460.2 ml in the classical group, we estimated that a minimum of 42 patients, equally divided on both groups, were required to detect a significant difference between the 2 compared groups, with a 2-tailed *α* of 0.05 and a (1 − *β*) of 0.80. To compensate for loss to follow-up and dropouts (estimated to be around 20%), 50 patients were ultimately included to the trial.

### Statistical Analysis

Statistical analysis was performed using SPSS software version 17 (IBM Corp, Chicago, Ill.). Continuous variables were described as mean ± SD. Categorical variables were reported using percentages. Student’s *t* test for paired samples was used to detect differences in the means of continuous variables and Fisher exact test or chi-square test was used for processing categorical variables. *P* values <0.05 were considered to be significant.

## RESULTS

### Preoperative Patients’ Characteristics

**Fig. 6.**
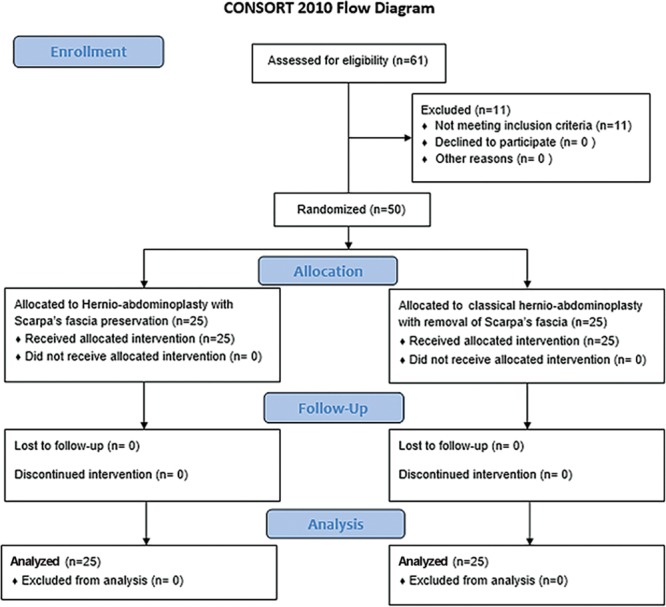
Consort flow chart illustrating the process of patient selection and exclusion.

After initial assessment of 61 patients, 11 patients did not meet the inclusion criteria of the study and were excluded and 50 patients were ultimately enrolled to the trial as illustrated in the Consort flow chart (Fig. 6).

Patients were 49 (98%) female and 1 (2%) male. The mean age of patients was 40.1 ± 7.7 years (range 28–58 years) and the mean BMI was 36.5 ± 1.6 kg/m^2^ (range 33–39 kg/m^2^). The mean preoperative waist circumference was 114.6 ± 4.6 cm (range 103–121 cm).

Ten patients (20%) had previous surgery for ventral hernia repair and 44 patients (88%) had previous abdominal surgery for indications other than hernia (19 cesarean section, 8 appendectomy, 8 laparoscopic cholecystectomy, 5 sleeve gastrectomy, and 4 hysterectomy).

Twenty-seven (54%) patients presented with umbilical hernia, 14 (28%) with epigastric hernia, and 9 (18%) with incisional hernia. The incisional hernias were following Pfannenstiel incision (n = 6) and lower midline incision (n=3). Twenty-five patients had associated medical comorbidities (15 diabetes mellitus and 10 hypertension) and 3 patients were smokers who quit smoking for 6 weeks before surgery.

There were no significant differences between the 2 groups in terms of patients’ age, gender distribution, weight, height, BMI, waist circumference, previous abdominal surgery, associated comorbidities, type of ventral hernia, and Pitanguy class as shown in Table 1.

**Table 1.**
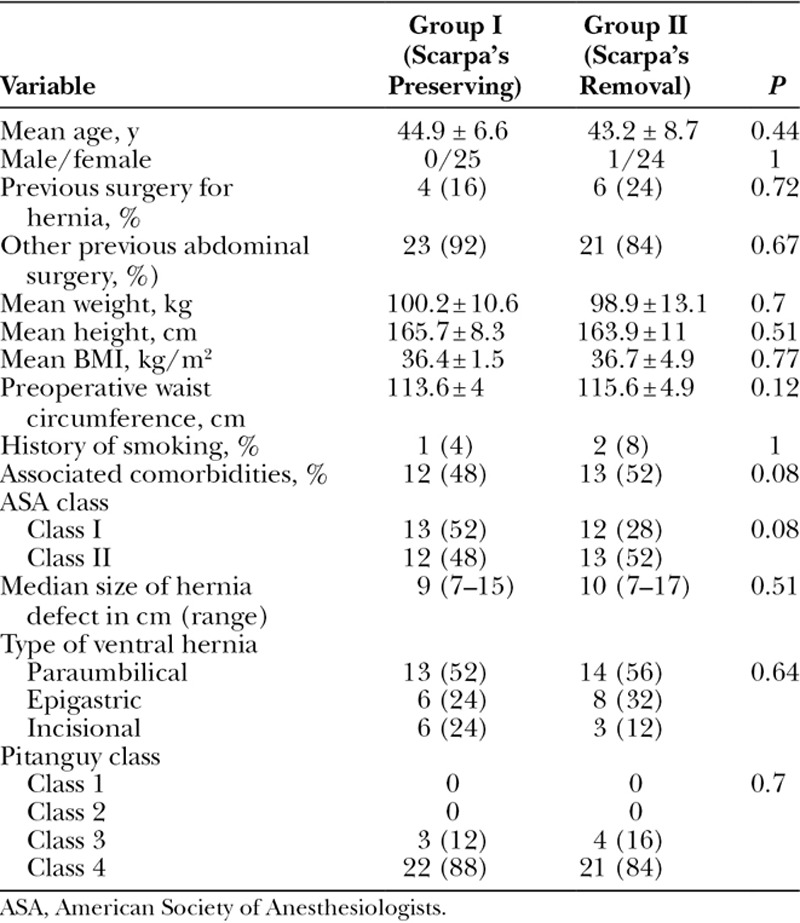
Preoperative Characteristics of Patients in Both Groups

### Postoperative Outcome

No significant difference in the operation time or hospital stay of both groups was observed. The median size of the abdominal wall defect was 9 cm (range 7–17 cm) as measured intraoperatively with the patient relaxed after tissue dissection. The median size of defect for umbilical hernias was 7 cm (range 7–9 cm), for epigastric hernia was 7 cm (range 7–10 cm), and for incisional hernia was 11 cm (range 9–17 cm).

Three patients (6%) experienced minor ischemia of the abdominal flap with no significant difference between the 2 groups regarding flap viability (96% versus 92%; *P* = 1).

In 13 patients, the hernia sac was found encroaching on the umbilical stalk and the umbilicus was excised, whereas in the remaining 37 patients the umbilicus was preserved. None of the patients with preserved umbilicus experienced umbilical gangrene on follow-up. Time to stitch removal in group I (18.4 ± 2.1 days) was similar to group II (18.3 ± 2.5 days) with no statistically significant difference (*P* = 0.88).

Both groups had comparable postoperative pain scores (6.9 ± 1 versus 7.2 ± 0.9; *P* = 0.27). The mean postoperative waist circumference in group I was significantly smaller than group II (111.2 ± 4 cm versus 114.7 ± 5 cm; *P* = 0.008). Patients in group I required significantly shorter time to return to work than group II (16.4 ± 2.3 versus 23.3 ± 3.8 days; *P* < 0.0001).

### Volume of Postoperative Drainage

The mean volume of postoperative wound drainage at 2, 4, 8, and 11 days postoperatively was significantly lower in group I than group II. The mean total volume of postoperative drainage was significantly lower in group I than group II (686 ± 183.5 versus 1410.8 ± 371.6 ml; *P* < 0.0001). Drains were removed earlier in group I compared with group II (11.6 ± 1.9 versus 20.5 ± 4.2 days; *P* < 0.0001; Tables 2, 3).

**Table 2.**
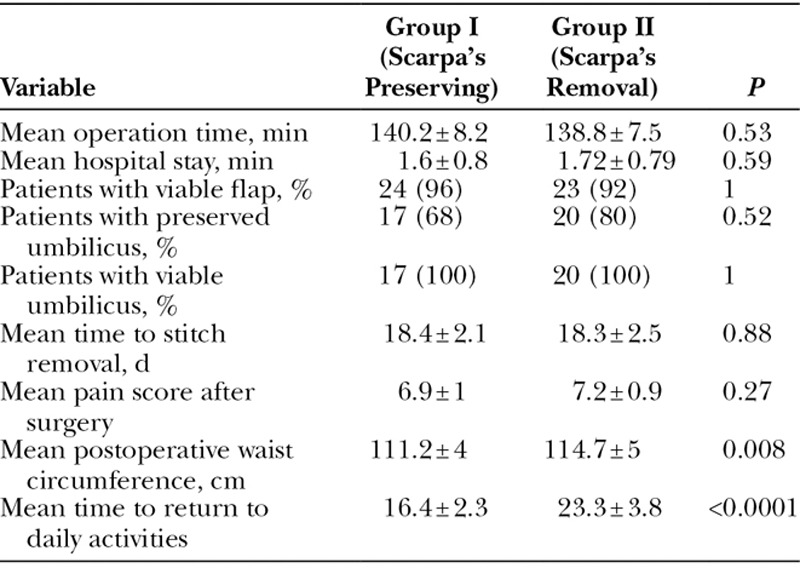
Postoperative Outcome of Patients in Both Groups

**Table 3.**
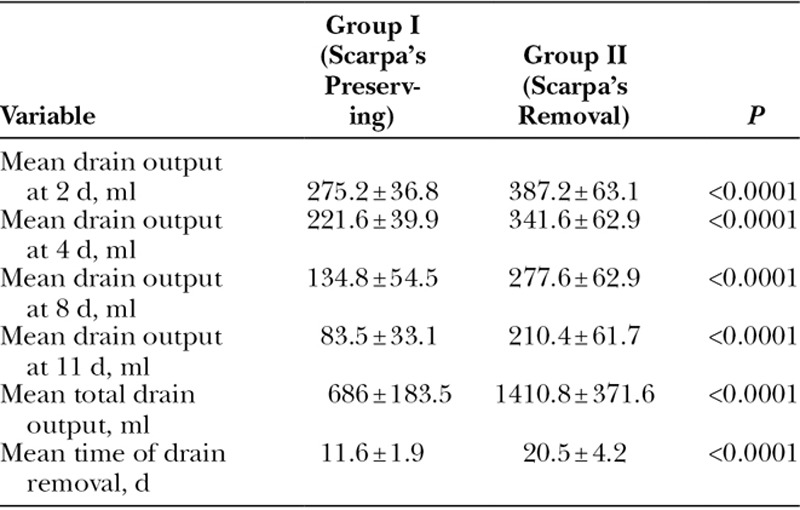
Volume of Postoperative Drainage at Different Time Points in Both Groups

### Postoperative Complications and Recurrence

Overall, 16 (32%) complications were recorded. Complications were all surgical site occurrences in the form of superficial surgical site infection of the skin and subcutaneous tissue that did not warrant mesh explantation or drainage (n = 5), minor flap disruption defined as partial dehiscence of the flap from its skin attachment due to ischemia or infection (n = 5) and seroma (n = 6). There were no recorded cases of hematoma or recurrence of ventral hernia after repair. No cases of skin necrosis were recorded. There was no significant difference between the 2 groups in regard to postoperative complications (24% versus 40%; *P* = 0.36; Table 4).

**Table 4.**
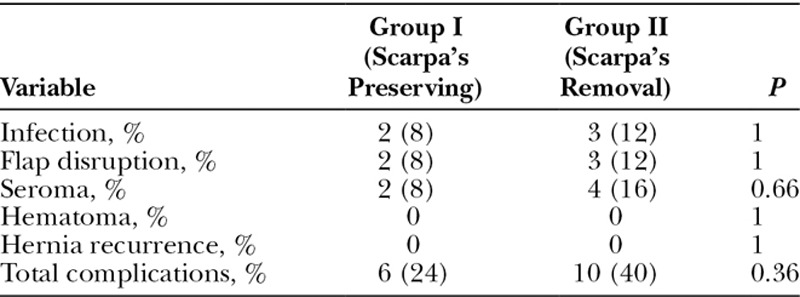
Postoperative Complications in Both Groups

### Patients’ Satisfaction and Quality of Life

There were no significant differences between the 2 groups regarding different domains of the Carolina’s equation questionnaire (Table 5). The overall patients’ satisfaction with the outcome of the procedure at 3 months postoperatively was higher in group I than group II, as 80% of group I patients were completely satisfied versus 48% in group II, whereas only 4% of group I patients were unsatisfied compared with 40% of group II patients (Table 6).

**Table 5.**
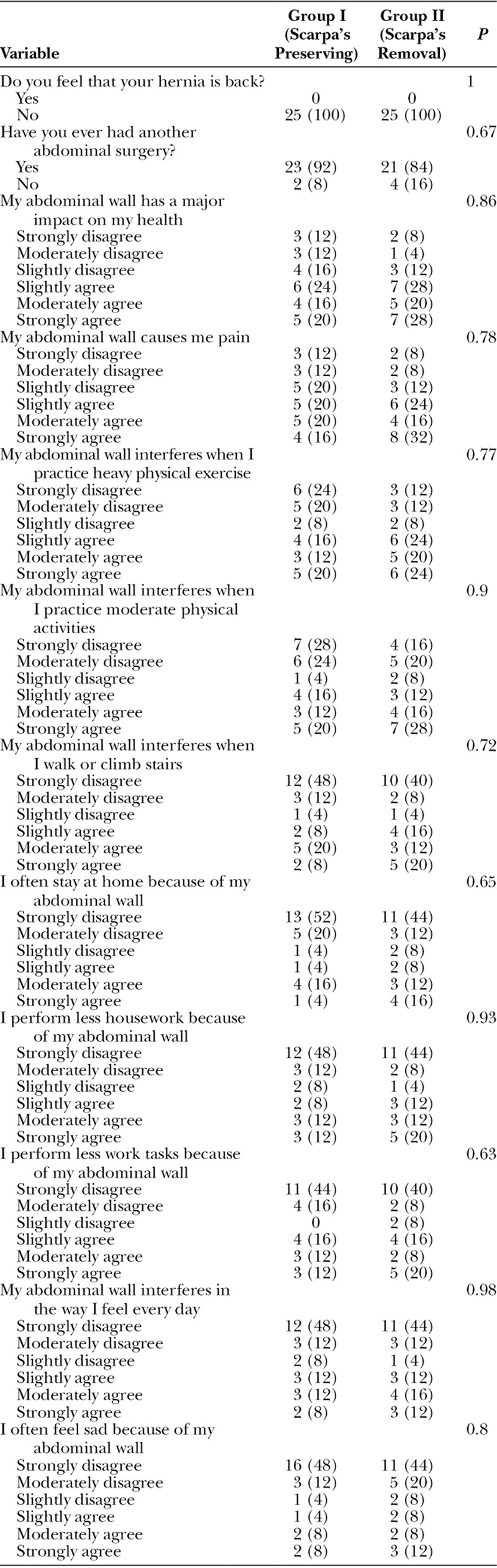
Postoperative Quality of Life According to Carolina’s Equation in Both Groups

**Table 6.**
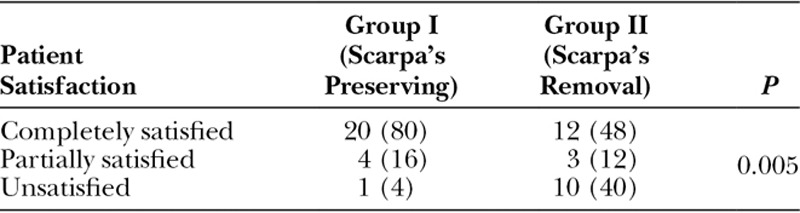
Patients’ Satisfaction with the Procedure in Each Group

## DISCUSSION

Patients with ventral hernias, who present with abdominal wall laxity and deformity, usually require combined surgical treatment of both problems as the repair of ventral hernias only may be associated with lower patients’ satisfaction. On the other hand, the good results obtained after combined ventral hernia repair and abdominoplasty usually have a positive impact on patient’s self-image and quality of life.^5,20^

Combining on-lay mesh repair of ventral hernia and abdominoplasty may, however, result in more seroma formation that requires prolonged treatment.^21^ Preservation of the Scarpa’s fascia may help mitigate this problem by preserving the arterio-veno-lymphatic system and maintaining reabsorption of the fluid released from interstitial spaces.^12^

Although previous studies^11,12,22,23^ assessed the efficacy of Scarpa’s fascia preservation in reducing seroma after abdominoplasty for abdominal wall deformity, none of these studies incorporated prosthetic ventral hernia repair with abdominoplasty. The present trial is the first to examine the impact of Scarpa’s fascia preservation in patients with ventral hernia who underwent combined hernio-abdominoplasty.

We excluded patients with major abdominal wall defects warranting abdominal wall reconstruction because preservation of the Scarpa’s fascia may not be feasible in these patients. We also excluded patients with complicated hernias because they usually warrant rapid intervention and carry higher risk of surgical site infection owing to the contaminated nature of the surgical field,^24^ which may negatively affect the outcome of the study.

The preservation of Scarpa’s fascia resulted in a significantly lower volume of postoperative wound drainage and earlier removal of drains. This was in concordance with the study by Costa-Ferreira et al.,^12^ who also documented similar findings in favor of the Scarpa’s fascia preservation group. It was notable that the total volume of drainage in either groups in our study was higher than that reported by Costa-Ferreira et al., perhaps the added element of on-lay prosthetic hernia repair in our trial contributed to this larger volume of drainage. Another plausible explanation of the larger drainage volume in our trial was the use of electrocautery for tissue dissection and elevation of the abdominal flaps and the higher BMI of patients.

Fang et al.^25^ also reported lower volume of drainage and shorter time required to remove drains in patients who underwent Scarpa’s fascia preserving abdominoplasty than patients who underwent classical abdominoplasty, which was associated with improved patient comfort and expedited recovery. It is worthy to highlight the controversy on the role of Scarpa’s fascia preservation because some surgeons advocated its preservation as a sponge layer to absorb lymphatic fluid; however, other authors considered this concept to have no anatomic or physiological foundation.^26–28^

The complication rate in both groups was 32%, which is higher than the complication rates (21%–24%) reported by other authors.^23,25^ No statistically significant difference in the complication rates was observed between the 2 groups in agreement with Fang et al.^25^ No cases of significant flap or umbilical necrosis were detected in either groups owing to meticulous preservation of the central and lateral blood supply of the flap.

In classical abdominoplasty, up to 30% of patients may develop seroma.^28^ The chief advantage of preserving the Scarpa’s fascia during abdominoplasty is reducing the incidence of seroma. Although the present trial found lower rate of seroma formation in the Scarpa’s fascia preservation group compared with the control group (8% versus 16%), this difference was not statistically significant. Similarly, in the study by Shahin et al.^23^ none of the patients with preserved Scarpa’s fascia developed seroma compared with 15% of patients in whom the Scarpa’s fascia was removed.

Scarpa’s fascia preservation also resulted in a significantly smaller waist circumference, in line with a previous study that concluded enhanced waistline in abdominoplasty with Scarpa’s fascia advancement.^29^ The medial advancement of the preserved Scarpa’s fascia on both sides results in medial traction of the whole superficial fascial system of the lower abdomen with tightening effect on the flanks, improving the waist, and obliterating the lower midline dead space.^30^

None of the patients in either groups experienced recurrence of ventral hernia on follow-up. Nonetheless, because the primary endpoint of the study included short-term outcomes such as the volume of postoperative drainage and incidence of seroma formation, the effect of Scarpa’s fascia preservation on hernia recurrence cannot be ascertained, as it requires longer follow-up.

Limitations of the present study include being a single-center study with relatively small number of patients in each group. Moreover, larger, multicenter trials including high-risk patients and patients with major abdominal wall defects warranting abdominal wall reconstruction are needed to reproduce the results of the present trial and examine the feasibility and outcome of Scarpa’s fascia preservation in other patient groups.

## CONCLUSIONS

Preservation of Scarpa’s fascia in combined ventral hernia repair and abdominoplasty was associated with significantly lower volume of postoperative drainage, earlier removal of drains, and smaller waist circumference compared with classical hernio-abdominoplasty with removal of Scarpa’s fascia.
